# Burnout and inter-role conflict in telework: a gendered resource erosion pathway via job crafting

**DOI:** 10.3389/fpsyg.2025.1746656

**Published:** 2026-01-14

**Authors:** Jesus Yeves, Laura Trujillo, Mariana Bargsted, Andy Luis Marrero-Vega

**Affiliations:** 1 Programa de Estudios Psicosociales del Trabajo (PEPET), Facultad de Psicología, Universidad Diego Portales, Santiago, Chile; 2 Núcleo Milenio sobre la evolución del trabajo (MNEW), Facultad de Psicología, Universidad Diego Portales, Santiago, Chile; 3 Programa de Doctorado de Ciencias de la Administración, Facultad de Administración y Economía, Universidad Diego Portales, Santiago, Chile; 4 Programa de Doctorado en Psicología, Facultad de Psicología, Universidad Diego Portales, Santiago, Chile

**Keywords:** burnout, family–work conflict, job crafting, telework, work–family conflict

## Abstract

**Introduction:**

While the impact of work–family conflict (WFC) and family–work conflict (FWC) on employee well-being is well documented, less is known about how burnout may, in turn, increase these conflicts through diminished self-regulatory capacity in a loss cycle. Through the lens of the Job Demands–Resources (JD-R) model and Conservation of Resources (COR) theory we propose that this association will be mediated by decreased job crafting. Gender differences in role expectations further can influence these dynamics, particularly in teleworking contexts where work and family boundaries blur. Therefore, the aim of our study is to investigate how job crafting mediates the relationship between burnout and inter-role conflicts (WFC/FWC) over time among teleworkers.

**Methods:**

A three-wave longitudinal design was conducted with 270 Chilean teleworkers (147 men, 123 women). Using cross-lagged panel models, we tested whether job crafting mediated the longitudinal association between burnout (T1) and inter-role conflicts (WFC/FWC) at T3, controlling prior levels of each variable. Subsequently, multi-group SEM analyses were performed to explore gender differences.

**Results:**

For male teleworkers, job crafting did not mediate the association between burnout and inter-role conflicts (WFC/FWC), however a direct association between burnout and WFC was observed over time. For female teleworkers, job crafting mediated the longitudinal association between burnout and FWC, but not the association between burnout and WFC. Moreover, no direct relationships were observed between burnout and either form of inter-role conflict over time.

**Conclusion:**

Job crafting may function as a self-regulatory buffer primarily for women, mitigating family-to-work interference under burnout. The findings suggest gendered, domain-specific resource erosion pathways and emphasize the need for targeted job crafting interventions to preserve resources and reduce work–family strain in remote work environments.

## 1 Introduction

Since the COVID-19 pandemic, the widespread use of communication technologies has transformed work, making telework, telecommuting, work-from-home, and hybrid models the dominant flexible work arrangements worldwide (Criscuolo et al., 2021; De Vincenzi et al., 2022; International Labour Organization (ILO), 2021). These shifts have given employees more flexibility and autonomy, which research shows can effectively decrease inter-role conflict between work and family demands (Yucel and Fan, 2023; Zakhem et al., 2022). Nonetheless, additional empirical evidence has indicated that dissolving spatial and temporal boundaries, in conjunction with the anticipation of “constant connectivity,” blurs the work–family interface, thereby heightening the risks of psychological distress and burnout (Elahi et al., 2022; Medina et al., 2021; Cheng et al., 2025). Hence, teleworkers may face intensified work–family conflict (WFC) when job demands intrude on their family domain, and family–work conflict (FWC) when family demands interfere with their work, compared to on-site workers. While the effects of WFC and FWC on job strain (e.g., burnout, psychological stress) have been examined (Brzykcy et al., 2024), research has largely overlooked the potential loss spiral whereby elevated strain amplifies job demands, such as role conflict, thereby perpetuating further strain (Hall et al., 2010; Golu et al., 2022). Moreover, in teleworking contexts, where certain demands may be intensified by the characteristics of this arrangement (Kumar et al., 2025), this loss cycle remains empirically untested. Furthermore, the role of self-undermining behaviors in this association, while it has been posited (Bakker et al., 2023), remains underexplored.

Workers experiencing high levels of burnout, a job strain measure characterized by emotional exhaustion, depersonalization, and reduced motivation (Maslach and Jackson, 1982), often feel depleted of the energy needed to engage in proactive work behaviors, such as job crafting, or adopt dysfunctional coping strategies (Bakker et al., 2023; Bakker and de Vries, 2020). Meta-analytic evidence indicates that job strain, implicating exhaustion and burnout, is negatively related to job crafting (Rudolph et al., 2017). Conceptualized within JD-R terms, job crafting involves proactively adjusting tasks and relationships to calibrate resources and demands, potentially reducing permeability between work and family domains (Lyu and Fan, 2022; Shi et al., 2022). Evidence also shows that by proactively adjusting resources and demands, employees can realign their work with personal circumstances, reducing WFC and FWC, and enhancing well-being (Tims et al., 2012; Petrou et al., 2018; Lyu and Fan, 2022). On the other hand, dysfunctional or maladaptive job crafting strategies, particularly those that blur work-family boundaries or focus solely on work, increase WFC and FWC (Zito et al., 2019). Therefore, we propose the following mediation process: burnout reduces the possibility of generating job crafting behaviors and leads to maladaptive coping strategies, which in turn increase WFC and FWC. However, the capacity to mobilize these self-regulatory adjustments may be conditioned by gendered constraints. In line with this sense, women may be more likely to implement proactive boundary protection and restorative tactics to manage family interference under strain (Lee and Riach, 2023), whereas men may exhibit different prioritization patterns, often focusing on reclaiming work-life boundaries (Lyu and Fan, 2022). These asymmetries suggest divergent resource erosion pathways, where the mediation through job crafting may be more critical for mitigating FWC among women, while men may experience a more direct spillover from burnout to WFC conflict.

Despite evidence that burnout reduces energy for proactive behaviors, such as job crafting, and that crafting can affect work–family permeability, several gaps still exist. First, this pathway has not been sufficiently tested in longitudinal studies, because most studies employ cross-sectional designs, which limit our ability to observe how loss spirals developing over time (Allen et al., 2019; Beckel et al., 2023). Second, the conditions under which the practice of crafting becomes maladaptive or counterproductive remain relatively underexplored in academic literature. Third, beyond traditional role orientations, gender differences in post-pandemic telework contexts remain under-examined as a proxy for differential household constraints and structural demands, despite well-documented variations in how men and women navigate remote work resources (Bianchi et al., 2012; Lyu and Fan, 2022). Finally, the consequences of telework post-COVID-19 remain underexplored, leaving unanswered questions about how new connectivity norms and boundary management affect loss spirals in both organizational and personal contexts.

To address these gaps, the present study employed a longitudinal design to investigate (1) how burnout influences job crafting, and (2) how these crafting behaviors in turn are expected to mediate the development of WFC and FWC among teleworkers. Additionally, we included gender not merely as a demographic variable, but as a proxy for differential structural constraints that may influence the specific prioritization and recovery strategies employees utilize to manage psychological strain and preserve role boundaries. By doing so, this research makes three key contributions. First, it provides longitudinal evidence of resource loss dynamics in remote work settings. Second, it elucidates the mediational mechanism of job crafting in the burnout–conflict sequence, offering a clearer understanding of when and how proactive coping succeeds or fails. Third, it reveals gender-specific patterns in these processes, informing the development of more tailored interventions and organizational policies that promote employee well-being and effective work–life integration.

## 2 Theoretical background and research hypothesis

### 2.1 The association between burnout and work–family conflict

Work–family conflict is commonly used as an umbrella term encompassing both directions of interrole conflict, defined as a situation in which “*the role pressures from the work and family domains are mutually incompatible in some respect*” (Greenhaus and Beutell, 1985, p. 77). WFC emerges when job demands intrude upon family responsibilities, whereas FWC arises when domestic obligations hinder work performance (Beckel et al., 2023). This bidirectional interference, from the role conflict perspective, stems from the fact that individuals have limited time and energy, and deciding where to devote these resources may cause distress and burnout when faced with such interference (Maertz and Boyar, 2011).

Research on flexible work arrangements, such as telework, has examined their dual potential to either exacerbate or mitigate WFC. Although initially regarded as a source of autonomy and flexibility for teleworkers, which therefore diminishes the interference (Gajendran and Harrison, 2007), later research on telework has shown that it creates permeability between roles, given that both work and family demands coexist in the same geographical location (Golden, 2011; Rahman and Singh, 2024). Moreover, due to telework blurring temporal boundaries, work obligations can extend beyond traditional office hours and intrude upon time typically reserved for family roles (Elahi et al., 2022).

In this study, we draw on both the Job Demands–Resources (JD-R) model and Conservation of Resources (COR) theory. Whereas JD-R provides a framework to conceptualize WFC and FWC as cross-domain demands that tax employees’ resources (Demerouti et al., 2001; Bakker and Demerouti, 2013), COR theory helps explain how sustained resource depletion may unfold over time and increase vulnerability to further demands (Hobfoll et al., 2018). From this perspective, persistent WFC and FWC require sustained effort and self-regulation and, when prolonged, can consume resources such as time, energy, and regulatory capacity, increasing strain and contributing to burnout symptoms such as emotional exhaustion (Haskić-Ožegović and Hadziahmetovic, 2023; Zhang et al., 2025), psychological distress, and other adverse health outcomes (Abdou et al., 2024; Borgmann et al., 2019). As resources become depleted, individuals may be less able to protect or replenish their resource pool, which can heighten exposure to subsequent demands and amplify stress reactions over time (Hobfoll et al., 2018).

A recent revision of the JD-R model (Bakker et al., 2023) proposed that employees may enter loss cycles when experiencing high levels of exhaustion, anxiety, or stress through undermining mechanisms. This aligns with Hobfoll et al. (2018) desperation principle, which posits that under high job strain, employees may resort to self-destructive strategies that paradoxically hinder performance. When employees experience burnout, prolonged exhaustion and reduced motivation erode their willingness or capacity to self-regulate, which in turn may lead to a lack of focus and work-related mistakes (Roczniewska and Bakker, 2021). Over time, self-undermining and dysregulation exacerbate hindrance demands by depleting personal resources and motivation.

Empirical research provides preliminary support for these loss-related dynamics. For example, Golu et al. (2022) found that burnout mediated the positive association between two job demands (work–family conflict; lack of equipment and supplies) and self-undermining behaviors. Similarly, Hall et al. (2010) also found evidence of a loss cycle in which job demands lead to work exhaustion, which in turn increased WFC.

We propose that burnout may intensify work–family conflict., by reducing employees’ self-regulatory capacity, weakening role boundaries, and promoting self-destructive strategies, thereby increasing the permeability between work and family domains.

*H1a*: Burnout at T1 is expected to be positively associated with subsequent increases in work-to-family conflict (WFC) over time.

*H1b*: Burnout at T1 is expected to be positively associated with subsequent increases in family-to-work conflict (FWC) over time.

### 2.2 The mediating role of job crafting

Previously, we have discussed how burnout may lead to an increase in work-to-family and family-to-work interference through self-undermining and self-destructive mechanisms. Job crafting literature offers a nuanced framework through which this association might be explained. In this sense, job crafting, a proactive behavior through which workers intend to adjust job characteristics to better match their individual preferences, motivations, and passions (Tims et al., 2012), may alleviate the impact of job demands on burnout over time (Bakker et al., 2023). This can be achieved through four strategies: increasing social job resources (e.g., social support, feedback), increasing structural resources (i.e., task-related resources), increasing challenging demands (i.e., those appraised as stimulating or likely to provide valuable outcomes), and decreasing hindrance demands (i.e., demands perceived as obstacles to goal achievement) (Tims et al., 2012).

Raghuram and Wiesenfeld (2004) proposed that virtual workers with higher tendencies to proactive behaviors, such as segmentation, will successfully construct boundaries to overcome the permeability between work and non-work domains. However, when employees experience chronic levels of burnout, i.e., extreme exhaustion or lose interest in their work, investing energy in these proactive behaviors becomes more difficult (Bakker et al., 2023). In a recent study, Song et al. (2025) found a negative association between job burnout and job crafting, also pondering a spillover effect of burnout from the workplace to the non-workplace. Prioritization through job crafting might be a valid alternative implemented by workers in this context, as indicated by the findings by Lyu and Fan (2022), where job crafting was found to reduce work-family interference, although with a differentiated gender effect. These findings support Hobfoll et al. (2018) conservation of resources theory and the loss cycles posited above through self-undermining behaviors from the JRD model (Bakker et al., 2023), as employees will perceive less energy and motivation and will seek to adjust their jobs to their new reality.

We posit that job crafting may serve as a mediator in the relationship between burnout and WFC/FWC, expected to function as a self-regulatory mechanism that teleworkers can implement under conditions of resource depletion.

*H2a*: Job crafting at T2 is expected to function as a self-regulatory mechanism linking burnout at T1 to later WFC at T3, such that burnout is expected to predict lower job crafting, which in turn is expected to predict higher WFC.

*H2b*: Job crafting at T2 is expected to function as a self-regulatory mechanism linking burnout at T1 to later FWC at T3, such that burnout is expected to predict lower job crafting, which in turn is expected to predict higher FWC.

### 2.3 The role of gender

While many studies have focused on the differentiated effects of WFC/FWC on burnout, relevant literature has paid less attention to the nuances of gender when dealing with burnout and how different strategies might be implemented to reduce job demands, especially in the context of telework (Escudero-Guirado et al., 2024). In this sense, gender differences in job demands are a crucial area of focus in contemporary workplace studies, especially given the traditional gender stereotypes that persist despite advancements in social gender awareness (Lyu and Fan, 2022). A time diary study revealed that women performed household labor at a rate 1.7 times greater than men (Bianchi et al., 2012). This disparity suggests that women may face a more significant impact from FWC, while men might experience greater effects from WFC. Nevertheless, investigations have produced inconsistent findings. In a meta-analysis, Shockley et al. (2017) showed that men and women do not significantly differ in their reports of WFC; however, a recent literature review (Escudero-Guirado et al., 2024) underscored the importance of hybrid and remote work opportunities for women, who might otherwise be forced to reduce their working hours due to their disproportionate responsibility for childcare and household duties.

Heras Recuero and Osca Segovia (2021) found that FWC did not predict emotional exhaustion among women, a finding that directly challenges gender roles theory (Eagly and Wood, 2012), which posits that women’s communal and caregiving orientations should heighten their vulnerability to family-induced strain. In another study, Lyu and Fan (2022) reported that WFC was unrelated to work engagement for either gender, while FWC significantly reduced women’s engagement, suggesting that women confront family interference by proactively reshaping their jobs. By contrast, men tended to prioritize reducing work’s intrusion into family life. Consistent with this pattern, Kim and Gong (2017) demonstrated that, when faced with WFC, female executives were more likely than their male counterparts to request flexible work arrangements, highlighting gender-specific job crafting strategies in response to inter-role conflict.

In contrast to the previously discussed findings, the role of gender in recovery experiences or self-regulation from a state of burnout remains underexplored in the literature. Lee and Riach (2023) studied the different responses from women in STEM fields (science, technology, engineering, and mathematics) to burnout and found a range of strategies for resource management implemented, i.e., through boundary-protecting actions (refusing overwork, setting limits, and confronting injustice), regenerative practices (taking breaks, cocooning, therapy, and identity reorientation), and promissory reinvestment tactics (changing roles or projects, mentoring others, and carefully pacing daily demands) to restore, conserve, and redistribute their energy without re-entering the cycle of depletion. By comparison, Kärkkäinen et al. (2017) reported that men were more likely to return to work after a sick leave due to burnout. Though the authors do not expand on these results, this may be evidence of gender differences in prioritization strategies.

The results indicate that gender may influence both the experience of inter-role conflict and subsequent recovery or adaptation to burnout; for women, distancing from work and pursuing flexible arrangements appear to be especially common strategies. Since female employees statistically face greater household demands than their male counterparts (Bianchi et al., 2012), and men and women tend to prioritize differently when confronted with family-work and work-family interference (Lyu and Fan, 2022), in a telework setting, we may expect a differentiated pattern to arise between female and male teleworkers. Despite mixed evidence, the available findings on gendered demands and prioritization strategies suggest that the burnout–job crafting–conflict pathway may differ by conflict direction. We propose the following hypotheses:

*H3*: Gender will moderate the indirect effect of burnout on FWC via job crafting, such that the indirect effect is expected to be stronger among women than among men.

*H4*: Gender will moderate the indirect effect of burnout on WFC via job crafting, such that the indirect effect is expected to be stronger among men than among women.

## 3 Materials and methods

### 3.1 Participants and procedures

Data were collected through an online panel managed by a market research company. Panel members were invited to participate in the study on the condition that they had been teleworking during the past month. Participants were informed that the survey was part of a funded project and were instructed to respond based on their recent work experience. The analytic sample was restricted to respondents reporting hybrid or fully remote work across all waves. The study was conducted in three waves: T1 data were collected in December 2023; T2, 4 months later (April 2024); and T3, 9 months after T1 (September 2024). Wave intervals were selected to support temporal ordering and allow sufficient time for substantive change, while limiting the influence of unobserved events that may occur over longer intervals (Menard, 2008).

A total of 567 participants completed the survey at T1 (49.6% male; mean age = 41.05, SD = 8.15). At T2, 437 individuals (51.5% male; mean age = 41.21, SD = 8.01) responded to the survey, representing 77.1% of the initial sample. At T3, 311 participants (52.4% male; mean age = 41.20, SD = 7.99) completed the questionnaire, corresponding to 54.9% of the original sample. Of these, 270 cases were retained for analysis after excluding participants with missing data on key study variables. Across the three waves, most participants reported engaging in hybrid telework (T1: 64.2%; T2: 66.1%; T3: 60.8%). In addition, the majority consistently indicated family responsibilities (T1: 65.3%; T2: 64.5%; T3: 62.7%). Independent-samples *t*-tests were conducted to examine potential baseline (T1) differences between participants who remained in the study and those who dropped out at later waves. Results revealed no significant differences between completers and dropouts from T1 to T2 in work-to-family conflict, family-to-work conflict, burnout, or job crafting (all *ps* > 0.25, |*d*| < 0.20). Similarly, no significant differences were observed between participants who completed all three waves and those who dropped out before T3 in any baseline variable (all *ps* > 0.50, |*d*| < 0.10). These results indicate that attrition was minimal and unsystematic, suggesting that data were missing at random and unlikely to bias the longitudinal estimates.

Multivariate normality was examined following the recommendations of Byrne (2010), Hair et al. (2010), and Kline (2016), according to which absolute values of skewness and kurtosis below 2 and 7, respectively, are acceptable. Skewness values for WFC (T1: 0.09; T2: 0.05; T3: 0.03), FWC (T1: 0.30; T2: 0.26; T3: 0.17), job crafting (T1: –0.95; T2: –0.75; T3: −0.71), and burnout (T1: –0.05; T2: –0.11; T3: 0.04) were all below 2. Kurtosis values for WFC (T1: –0.82; T2: –0.83; T3: –0.84), FWC (T1: –0.77; T2: –0.82; T3: −0.91), job crafting (T1: 2.79; T2: 2.81; T3: 1.45), and burnout (T1: –0.59; T2: –0.65; T3: −0.37) were all below 7.

### 3.2 Measures

Burnout was assessed using a shortened version of the Maslach Burnout Inventory (MBI), based on the Chilean adaptation validated by Olivares-Faúndez et al. (2014), whose full version comprises 22 items. For this study, we selected 9 representative items, three from each subscale: Emotional Exhaustion (EE), Depersonalization (DP), and Personal Accomplishment (PA); to reduce respondent burden while preserving conceptual coverage. All items were rated on a 5-point Likert scale. Internal consistency was satisfactory across the three measurement waves (T1 *α* = 0.84; T2 α = 0.85; T3 α = 0.86).

Job Crafting. The short version of the Job Crafting Scale developed by Tims et al. (2012) and validated in Spanish by Sora et al. (2018) was used. The scale consists of 12 items rated on a 5-point Likert scale, covering four dimensions: increasing structural job resources, decreasing hindering job demands, increasing social job resources, and increasing challenging job demands. Cronbach’s alpha indicated good reliability at all three time points (T1 α = 0.81; T2 α = 0.80; T3 α = 0.80).

Work–Family Conflict (WFC) and Family–Work Conflict (FWC). These variables were assessed using a shortened version of the Survey Work-Home Interaction–Nijmegen (SWING) questionnaire, originally developed by Geurts et al. (2005) and adapted into Spanish by Moreno Jiménez et al. (2009). A total of 6 items were used: 3 assessing negative work-to-family interaction (WFC) and 3 assessing negative family-to-work interaction (FWC). Responses were rated on a 5-point Likert scale. Internal consistency was acceptable and stable across time points (WFC: T1 α = 0.84; T2 α = 0.83; T3 α = 0.83; FWC: T1 α = 0.90; T2 α = 0.91; T3 α = 0.91).

Control Variable. The number of dependents was included as a control variable. This measure was calculated by summing the total number of individuals dependent on the respondent, including both minors and other dependents.

### 3.3 Analytical procedure

Descriptive statistics and correlations were computed for all observed variables across the three waves. Longitudinal structural relationships were examined using Structural Equation Modeling (SEM). To address concerns related to model complexity, we followed the recommendations of Bentler and Chou (1987) and Harris and Schaubroeck (1991), who advise limiting the number of observed indicators to approximately 20 to reduce the likelihood of improper solutions and improve model fit. Therefore, we constructed composite variables for WFC, FWC, job crafting, and burnout, which were used as observed indicators in the structural model.

Before estimating the structural models, longitudinal confirmatory factor analyses (CFAs) were conducted to evaluate the measurement structure of each latent construct across the three waves. To examine whether the structural relationships differed by gender, we conducted a multigroup crosslagged panel model analysis using AMOS 24. We first estimated an unconstrained model, allowing all parameters (regression paths) to vary freely between groups. The standardized path coefficients (betas) obtained from this model were used to interpret group-specific relationships. Next, we tested for structural invariance by sequentially comparing increasingly constrained models: the structural weights model, in which regression paths were constrained to be equal across groups, and the structural residuals model, in which error variances were also constrained to equality. Model comparisons were evaluated using differences in comparative fit index (ΔCFI), as recommended by Cheung and Rensvold (2002), with a ΔCFI ≤ 0.01 indicating invariance.

Model fit was evaluated using conventional indices, root mean square error of approximation (RMSEA), comparative fit index (CFI), and standardized root mean square residual (SRMR), according to established guidelines (Kline, 2016). Values indicative of good (or acceptable) model fit are: RMSEA < 0.06 (0.08), CFI > 0.95 (0.90), GFI > 0.95 (0.90), TLI > 0.95 (0.90), and SRMR < 0.08 (0.09).

## 4 Results

Table 1 presents the means, standard deviations, and correlations among the study variables. On average, participants reported moderate levels of work-to-family conflict (WFC), family-to-work conflict (FWC), and burnout across the three measurement waves, as well as relatively high levels of job crafting. Correlations among the same constructs across waves were strong and positive, indicating temporal stability for WFC (*r*s = 0.85–0.91), FWC (*r*s = 0.90–0.94), and burnout (*r*s = 0.73–0.87). Although the number of dependents did not show significant zero-order correlations with the main study variables, it was retained as a control variable given its theoretical relevance for work–family dynamics.

**Table 1:** Means, standard deviations, and correlations among study variables (N = 270).

Variable	M	SD	1	2	3	4	5	6	7	8	9	10	11	12	13
1. Gender	0.54	0.50													
2. Number of dependents	1.17	1.24	0.08												
3. WFC T1	2.85	1.08	−0.05	−0.07											
4. WFC T2	2.86	1.08	−0.06	−0.01	0.91***										
5. WFC T3	2.84	1.10	−0.07	−0.06	0.85***	0.90***									
6. FWC T1	2.49	0.94	−0.05	−0.03	0.53***	0.53***	0.51***								
7. FWC T2	2.52	0.96	−0.05	−0.02	0.49***	0.53***	0.48***	0.94***							
8. FWC T3	2.52	0.96	−0.04	−0.01	0.46***	0.50***	0.52***	0.90***	0.93***						
9. Burnout T1	2.47	0.73	−0.02	−0.05	0.53***	0.49***	0.51***	0.45***	0.43***	0.44**					
10. Burnout T2	2.47	0.75	−0.05	−0.03	0.49***	0.53***	0.49***	0.45***	0.46***	0.49***	0.87***				
11. Burnout T3	2.48	0.72	−0.08	−0.04	0.41***	0.45***	0.41***	0.38***	0.37***	0.40***	0.73***	0.85***			
12. Job crafting T1	3.68	0.59	−0.05	0.03	−0.12*	−0.07	−0.14*	−0.08	−0.11	−0.14*	−0.33***	−0.25***	−0.19**		
13. Job crafting T2	3.61	0.55	−0.09	−0.01	−0.15*	−0.19**	−0.17**	−0.11	−0.11	−0.16**	−0.31***	−0.42***	−0.33***	0.70***	
14. Job crafting T3	3.52	0.63	−0.11*	0.03	−0.08	−0.13*	−0.13*	−0.08	−0.09	−0.11	−0.22***	−0.23***	−0.18**	0.27***	0.49***

WFC, Work-to-family conflict; FWC, Family-to-work conflict. Gender is coded as 0 = Female, 1 = Male. *p* < 0.05, *p < 0.01, **p < 0.001.

We evaluated longitudinal configural invariance for each latent construct measured at three waves. Given that the WFC and FWC scales consisted of only three items each and their individual CFA models were just-identified, measurement invariance could not be formally examined for each construct separately. Instead, both scales were combined into a partial measurement model to permit invariance testing. As shown in Table 2, all constructs demonstrated strong longitudinal measurement invariance across the three waves. For *burnout* and *job crafting*, model fit was excellent at all levels of invariance (CFI and TLI ≥ 0.97, RMSEA ≤ 0.06), and ΔCFI values remained below 0.01, indicating full configural, metric, scalar, and strict invariance. Similarly, the combined WFC and FWC model showed acceptable fit, with notable improvement in fit indices at higher levels of invariance (ΔCFI ≤ 0.006). Structural invariance models yielded equivalent or improved fit for all constructs, supporting the longitudinal stability of both measurement and structural relations over time.

**Table 2:** Longitudinal measurement invariance of the study constructs.

Construct	Model	*χ*^2^(df)	CFI	TLI	RMSEA	SRMR	ΔCFI
Burnout	Configural	116.796 (72)	0.998	0.996	0.048	0.030	—
	Metric	124.547 (84)	0.998	0.997	0.042	0.031	0.000
	Scalar	151.988 (132)	0.999	0.999	0.024	0.030	0.001
	Estric	151.988 (132)	0.999	0.999	0.024	0.030	0.000
Job Crafting	Configural	279.359 (144)	0.985	0.971	0.063	0.044	—
	Metric	287.898 (160)	0.987	0.984	0.055	0.047	0.002
	Scalar	331.612 (224)	0.989	0.990	0.043	0.045	0.002
	Estric	331.612 (224)	0.989	0.990	0.043	0.045	0.000
WFC + FWC	Configural	180.465 (24)	0.971	0.946	0.156	0.054	—
	Metric	170.250 (32)	0.975	0.964	0.127	0.056	0.004
	Scalar	169.557 (64)	0.981	0.986	0.078	0.054	0.006
	Estric	169.557 (64)	0.981	0.986	0.078	0.054	0.000

WFC, work-to-family conflict; FWC, family-to-work conflict.

### 4.1 Hypothesis testing

Hypotheses 1 and 2 proposed that burnout would be positively related to work–family conflict (WFC; H1a) and family–work conflict (FWC; H1b) over time, and that job crafting at T2 would mediate the relationships between burnout at T1 and WFC (H2a) and FWC (H2b) at T3. To test these hypotheses, two structural equation models (SEMs) were estimated for the overall sample, combining men and women, considering WFC and FWC as separate outcomes.

The general SEM for WFC showed excellent fit to the data (*χ*^2^(22) = 38.91, *χ*^2^/df = 1.77, GFI = 0.972, CFI = 0.992, TLI = 0.984, RMSEA = 0.053, 90% CI [0.024, 0.080]). Burnout, job crafting, and WFC exhibited strong temporal stability across the three waves (*β*s = 0.85–0.91, *p* < 0.001). Burnout at T1 positively predicted WFC at T3 (*β* = 0.09, *p* = 0.003), supporting H1a. However, the indirect effect through job crafting was not significant, as burnout did not predict job crafting at T2 (*β* = −0.07, *p* = 0.18), and job crafting did not predict WFC at T3 (*β* = 0.02, *p* = 0.50). Therefore, H2a was not supported. A small positive association was also observed between the number of dependents and WFC at T2 (*β* = 0.05, *p* = 0.039), suggesting that greater family demands slightly increased subsequent WFC.

The general SEM for FWC showed acceptable fit (*χ*^2^(22) = 58.34, *χ*^2^/df = 2.65, GFI = 0.960, CFI = 0.984, TLI = 0.968, RMSEA = 0.078, 90% CI [0.054, 0.103]). Burnout, job crafting, and FWC demonstrated strong temporal stability across waves (*β*s = 0.86–0.94, *p* < 0.001). Cross-lagged effects from burnout at T1 to job crafting at T2 were nonsignificant (*β* = −0.08, *p* = 0.09), whereas job crafting at T2 negatively predicted FWC at T3 (*β* = −0.06, *p* = 0.008). The direct effect of burnout on FWC (*β* = 0.03, *p* = 0.32) was nonsignificant. Consequently, H1b and H2b were not supported in the overall sample, although job crafting appeared to play a protective role by reducing subsequent levels of FWC.

Hypotheses 3 and 4 proposed that the indirect effect of burnout on FWC through job crafting would be stronger among women than men (H3), and that the indirect effect of burnout on WFC through job crafting would be stronger among men than women (H4). To examine these moderation effects by gender, multi-group structural equation models were estimated for men (n = 147) and women (n = 123). Overall fit for the unconstrained multi-group models was acceptable for both outcomes. For WFC, fit indices were: *χ*^2^(44) = 97.96, *χ*^2^/df = 2.23, GFI = 0.933, CFI = 0.977, TLI = 0.953, RMSEA = 0.068 (90% CI [0.050, 0.087]). For FWC, fit indices were: *χ*^2^(44) = 81.32, *χ*^2^/df = 2.67, GFI = 0.923, CFI = 0.986, TLI = 0.972, RMSEA = 0.056 (90% CI [0.037, 0.075]). When structural weights were constrained to be equal across men and women, model fit decreased significantly for both outcomes (WFC: Δ*χ*^2^(10) = 20.44, *p* < 0.05; FWC: Δ*χ*^2^(10) = 39.43, *p* < 0.001), indicating that some structural paths differed by gender.

**Figure 1:**
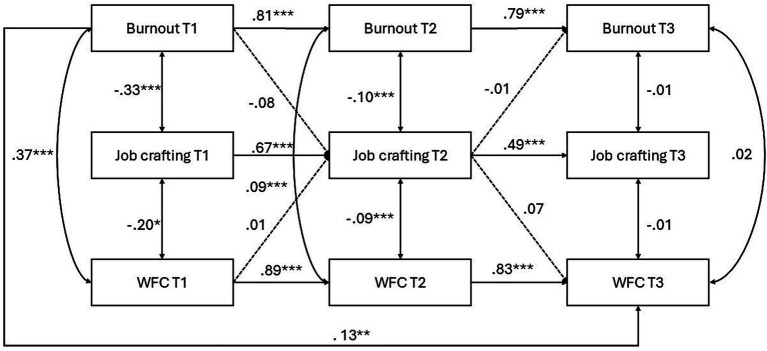
Longitudinal model of burnout, job crafting, and WFC for men. Standardized coefficients are shown. ***p < 0.001, **p < 0.01, *p < 0.05.

For WFC, the model was estimated separately for men and women. Across both groups, autoregressive effects were consistently strong. Among men (Figure 1), burnout at T1 significantly predicted burnout at T2 (*β* = 0.81, *p* < 0.001), and burnout at T2 predicted burnout at T3 (*β* = 0.80, *p* < 0.001). Similarly, job crafting showed significant stability from T1 to T2 (*β* = 0.67, *p* < 0.001) and from T2 to T3 (*β* = 0.50, *p* < 0.001), and WFC displayed strong temporal stability from T1 to T2 (*β* = 0.89, *p* < 0.001) and from T2 to T3 (*β* = 0.88, *p* < 0.001). A direct positive association emerged between burnout at T1 and WFC at T3 (*β* = 0.13, *p* < 0.01), partially supporting H1a. However, the indirect pathway through job crafting (H2a) was not supported.

**Figure 2:**
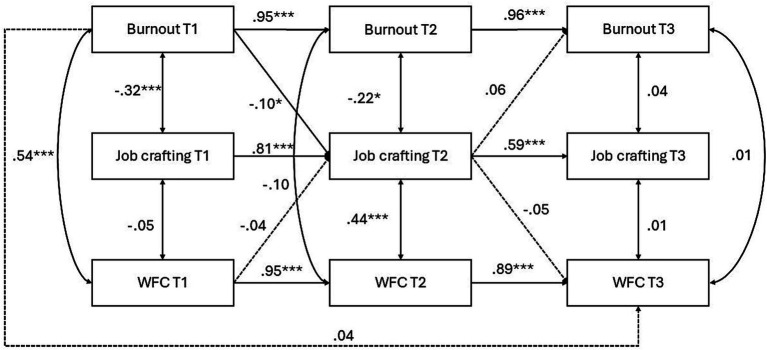
Longitudinal model of burnout, job crafting, and WFC for women. Standardized coefficients are shown. ***p < 0.001, **p < 0.01, *p < 0.05.

Among women (Figure 2), autoregressive effects were also strong across both intervals. Burnout at T1 predicted burnout at T2 (*β* = 0.95, *p* < 0.001), and burnout at T2 predicted burnout at T3 (*β* = 0.94, *p* < 0.001). Job crafting showed significant stability from T1 to T2 (*β* = 0.79, *p* < 0.001) and from T2 to T3 (*β* = 0.48, *p* < 0.001). WFC was also stable over time (from T1 to T2, *β* = 0.93, *p* < 0.001; and from T2 to T3, *β* = 0.87, *p* < 0.001). Among the cross-lagged effects, only the path from burnout at T1 to job crafting at T2 was negatively significant (*β* = −0.10, *p* < 0.05), whereas the direct effect of burnout at T1 on WFC at T3 was nonsignificant (*β* = 0.04, ns). Thus, H1a and H2a were not supported for women, and the expected stronger indirect effect of burnout on WFC via job crafting among men (H4) was not supported.

**Figure 3:**
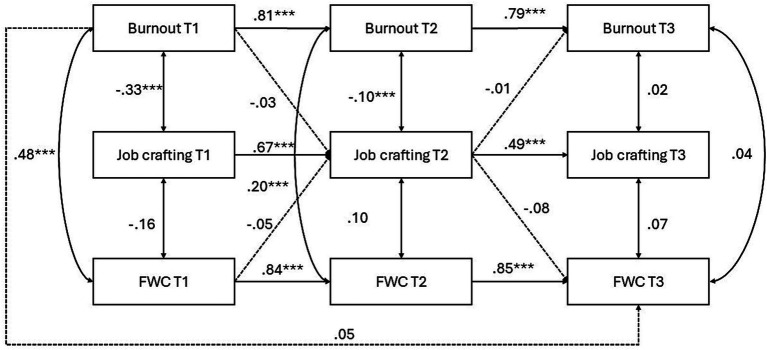
Longitudinal model of burnout, job crafting, and FWC for men. Standardized coefficients are shown. ***p < 0.001, **p < 0.01, *p < 0.05.

For FWC, the model was again tested by gender. Among men (Figure 3), autoregressive effects were again strong across both intervals. Burnout at T1 predicted burnout at T2 (*β* = 0.81, *p* < 0.001), and burnout at T2 predicted burnout at T3 (*β* = 0.80, *p* < 0.001). Job crafting showed significant stability from T1 to T2 (*β* = 0.67, *p* < 0.001) and from T2 to T3 (*β* = 0.50, *p* < 0.001). FWC was also stable from T1 to T2 (*β* = 0.84, *p* < 0.001) and from T2 to T3 (*β* = 0.85, *p* < 0.001). However, cross-lagged paths were nonsignificant, and no evidence of mediation emerged. Therefore, H1b and H2b were not supported for men.

**Figure 4:**
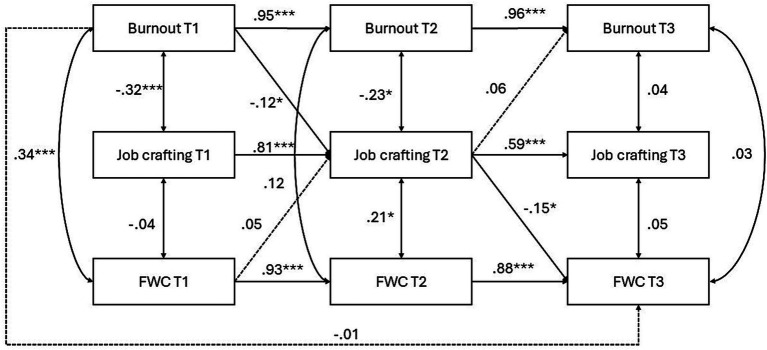
Longitudinal model of burnout, job crafting, and FWC for women. Standardized coefficients are shown. ***p < 0.001, **p < 0.01, *p < 0.05.

Among women (Figure 4), autoregressive effects were again strong across both intervals. Burnout at T1 predicted burnout at T2 (*β* = 0.95, *p* < 0.001), and burnout at T2 predicted burnout at T3 (*β* = 0.94, *p* < 0.001). Job crafting showed significant stability from T1 to T2 (*β* = 0.81, *p* < 0.001) and from T2 to T3 (*β* = 0.48, *p* < 0.001). FWC was also stable over time (T1 → T2, *β* = 0.93, *p* < 0.001; T2 → T3, *β* = 0.87, *p* < 0.001). Importantly, burnout at T1 negatively predicted job crafting at T2 (*β* = −0.12, *p* = 0.03, 90% CI [−0.21, −0.01]), and job crafting at T2 was negatively associated with FWC at T3 (*β* = −0.15, *p* = 0.02, 90% CI [−0.16, −0.02]). The indirect effect of burnout at T1 on FWC at T3 through job crafting at T2 was significant (bootstrap CI excluded zero). These results provide partial support for H1b and H2b, and support for H3, as the mediation effect was only observed among women.

## 5 Discussion

The present study aimed to examine, through a longitudinal design, how job crafting mediates the relationship between burnout and WFC and FWC among teleworkers, considering gender as a moderator. Contrary to our initial hypothesis, baseline burnout did not initiate a resource loss cycle via job crafting that would subsequently influence WFC or FWC. However, moderation analyses suggested a more nuanced perspective on resource erosion. For instance, among male participants, job crafting did not function as a significant mediator for either WFC or FWC, implying that under high psychological strain men may be less likely to enact proactive adjustments to mitigate demands. Notably, we observed a significant direct association between burnout (T1) and WFC (T3) for men, indicating that elevated burnout predicts greater interference from work to family even over extended intervals. This finding aligns with prior research (Eagly and Wood, 2012) suggesting that men may experience greater spillover from work strain into the family domain, possibly due to differences in role salience or coping strategies.

While no significant partial mediating effect was found for men, among women, we found that job crafting mediated the association between burnout and FWC. In line with our hypotheses, higher burnout lead to a decrease in job crafting behaviors, for female participants. Results suggested that women experiencing high levels of psychological strain at work will engage in fewer strategies to mitigate the effects of their job demands. This may be due to the fact that workers experiencing high levels of burnout may often feel depleted of the energy needed to engage in job crafting activities (Bakker et al., 2023). What is most interesting here, is that in this study, we only found this relation to be significant for women. Overall, H2 was partially supported, with mediation occurring only in the female subsample, and specifically in relation to family-to-work conflict. These results lead us to highlight the relevance of conducting studies where gender is not a control variable, but a relevant variable when it comes to understanding the potential impact of burnout on inter-role conflict and how job crafting may or may not contribute to reducing that impact. These findings are further reinforced by Hypothesis 3, which proposed that the indirect effect of burnout on FWC via job crafting would be stronger among women than men. In this case, the results indicate that women’s proactive behaviors, or their reduction under high burnout, play a more central role in shaping family-to-work interference. Hypothesis 4, which proposed that the indirect effect of burnout on WFC via job crafting would be stronger among men than women, was not supported. Among male participants, burnout did not significantly reduce job crafting, nor did job crafting buffer the effects of burnout on work-to-family conflict.

Taken together, these findings suggest that the direct positive link between burnout and conflict is more evident for men in the WFC direction, whereas for women, the association with FWC is more complex and operates through self-regulatory processes such as job crafting. Rather than men, women may rely more heavily on job crafting as a mechanism to mitigate the spillover of work demands into the family domain. This pattern may reflect gendered differences in boundary management and resource sensitivity, whereby women, often facing greater cumulative demands across work and family spheres, are more vulnerable to resource depletion and, consequently, more dependent on proactive strategies to preserve balance and reduce conflict (Escudero-Guirado et al., 2024).

Consistent with our findings, López-Madrigal et al. (2021) found that women were more prone to engage in coping strategies, as opposed to men. Further qualitative evidence from STEM contexts shows that women commonly mobilize proactive resource-management tactics after burnout, tightening boundaries around availability, engaging in restorative practices (e.g., “cocooning,” therapy), and selectively reinvesting in roles or projects, aimed at rebuilding capacity and preventing renewed loss cycles (Lee and Riach, 2023). By contrast, prospective evidence from a mixed-sector Finnish cohort indicates that men have a higher probability of returning to work after burnout was clinically diagnosed (Kärkkäinen et al., 2017). These studies align with a gendered asymmetry whereby men’s burnout may more readily translate into WFC through a direct pathway, while women’s responses are more likely to involve deliberate self-regulatory efforts to buffer family-directed spillover.

Although loss cycle has been scarcely studied, existing research has often focused on specific contextual situations, making generalizations difficult. Results from Golu et al. (2022), for instance, while promising, were obtained during the pandemic and from a sample of healthcare workers. The uncertainty and particular demands from this context should be considered. Hall et al. (2010) presented a solid methodology, accounting for longitudinal dynamics and within/between analysis. Yet, their sample of police officers also raises the question of generalization. Our study is likewise contextually bounded, given the focus on remote workers. Nonetheless, it might provide evidence of a pattern in loss cycles specific to this practice, where men do not experience resource depletion through self-undermining behaviors (or, in this case, through a decrease in job crafting behaviors), but through a direct path that occurs over an extended period of time.

### 5.1 Theoretical implications

The findings yield significant theoretical implications by integrating COR theory (Hobfoll and Freedy, 1993) and the JD-R model (Bakker et al., 2023) to explain gendered resource erosion pathways. By synthesizing the COR perspective on stressor-induced resource depletion with the JD-R model’s focus on proactive behaviors, our results illustrate how burnout diminishes women’s job crafting, a key self-regulatory mechanism, thereby intensifying FWC as individuals lose the capacity to mobilize protective resources. This highlights that the loss cycles posited by the latest JD-R revision are triggered by a burnout-induced decrease in crafting, which subsequently increases job demands over time. Crucially, these mechanisms are gender-specific; while women face an indirect erosion of resources, men appear to experience a more direct path of WFC. Furthermore, this framework underscores that while telework-specific resources like job autonomy can mitigate conflict, the effectiveness of these resources depends on the individual’s remaining energy to engage in proactive job design.

Related to this, gender-differentiated patterns underscore another relevant theoretical implication. Specifically, women’s proactive investment in job crafting appears more critical for mitigating family-to-work interference, likely reflecting greater cumulative caregiving demands and more permeable work–family boundaries in telework settings. In contrast, for men, the dominant pathway seems to be a direct work-to-family spillover, whereby burnout leads to increased WFC, independent of resource-regulatory mechanisms such as job crafting. These insights highlight a relevant metatheoretical need to integrate a gender perspective into the theories and models commonly used to explain the relationships between burnout, work–family conflict, and family–work conflict. Incorporating gender as a core analytical dimension would allow for a more comprehensive understanding of how gender differences shape individuals’ experiences and behaviors at work. By doing so, future research could move beyond gender-neutral assumptions and better capture the ways in which social expectations and normative roles influence both the experience and the expression of strain in the work–family interface.

### 5.2 Practical implications

Regarding implications for practitioners, we suggest that organizations implement targeted job crafting interventions. For women, in particular, training should emphasize increasing structural and social resources and decreasing hindering demands, such as renegotiating response-time norms, clarifying role priorities, and instituting “no-meeting” blocks, to buffer FWC when burnout is elevated (Escudero-Guirado et al., 2024). These interventions should be framed as ongoing, team-supported practices rather than one-off workshops, with periodic follow-ups to sustain gains.

Providing concrete segmentation tactics (e.g., clearly defined start/stop rituals and protected deep-work windows) alongside family-supportive supervisor behaviors can reduce WFC (Mergener et al., 2025). This is particularly relevant for men, who showed a more direct burnout-to-WFC pathway, and may benefit from explicit, manager-endorsed limits on availability and cross-domain intrusions. Telework job design should also be calibrated to prevent strain from spilling over into the home domain. Recommended practices include aligning workload with autonomy, formalizing availability norms, restricting after-hours communications, and deploying technology-enabled boundary controls (e.g., delayed-send defaults, clear status policies).

In addition, to sustain resource “caravans,” organizations should adopt resource equity and care-sensitive policies. Flexible scheduling, dependable backup care, caregiving leave, and fair task allocation during peak demand periods help preserve core resources and create the conditions under which job crafting can be effective for both women and men (Hobfoll, 2001). Attention to intersectional caregiving responsibilities will further enhance policy impact. Finally, manager enablement is critical. Leaders should be equipped to recognize early indicators of resource depletion and to co-create local crafting opportunities, such as task reprioritization, simplified workflows, and peer coaching, while remaining attentive to gendered constraints in access to time, support, and visibility (Wu et al., 2025). Embedding these practices into routine management cycles (e.g., weekly check-ins, quarterly workload reviews) will promote durable gains in balance and performance.

### 5.3 Limitations and future research

Beyond our contributions, this study entails limitations that future research should address. First, considering that variables were measured via self-report, we acknowledge the risk of common method bias. Consistent with recommended procedural remedies to mitigate common method bias (Podsakoff et al., 2024), temporal separation was introduced by collecting the predictor, mediator, and outcomes across three waves rather than in a single survey. This design was intended to reduce respondents’ ability and motivation to rely on prior answers and to help support temporal ordering. In addition, we conducted Harman’s single-factor test separately for each wave using all self-report items measured at that wave in an unrotated exploratory factor analysis. In none of the waves did a single factor account for the majority of the variance (T1: 27.63%; T2: 28.15%; T3: 22.95%), suggesting that common method variance is unlikely to be a dominant concern. Second, while we modeled overall job crafting, theory suggests that its dimensions, increasing structural resources, increasing social resources, increasing challenging demands, and decreasing hindering demands, may differentially relate to WFC and FWC (Tims et al., 2012). The present pattern is most consistent with the resource-oriented facets (increasing resources; decreasing hindering demands) as protective against FWC, a nuance future work should test explicitly.

Moreover, subsequent research should account for contextual factors that were not tested in this study and may shape job crafting and work–family dynamics under teleworking arrangements. Relevant distinctions include full-time versus part-time telework, as well as working from home versus other remote locations, which may entail different levels of autonomy, social contact, and boundary permeability. Another important avenue concerns the voluntariness of telework, since choosing to telework versus being required to do so could influence the degree to which employees proactively craft their jobs. Likewise, the presence of other family members simultaneously teleworking may alter both resource availability and interference within the home environment. Future studies should incorporate additional explanatory variables, such as boundary management strategies, organizational support, or perceived control over working conditions, to better capture the complex mechanisms linking job crafting to work–family outcomes.

## 6 Conclusion

The longitudinal evidence shows that burnout is a robust antecedent of WFC over time, whereas its association with FWC is better understood through the lens of gender. Job crafting emerged as a protective mechanism that reduced subsequent FWC but did not account for the burnout–WFC link. This domain-specific pattern suggests that resource erosion from burnout more readily spills over into work interfering with family, while family demands can be buffered when employees actively reshape tasks, relationships, and hindering demands.

Gender-segmented analyses refined this picture. Among men, burnout exerted a direct effect on later WFC, consistent with a loss spiral that does not rely on intermediary self-regulatory behaviors. Among women, however, burnout undermined job crafting at the intermediate wave, and lower crafting predicted higher FWC at the final wave, indicating an indirect pathway. These results align with JD-R and COR perspectives, highlighting job crafting as a gender-sensitive buffer that preserves resources in the family-to-work domain.

Methodologically, the use of three waves, cross-lagged structural models, and tests of temporal stability supports the validity of the inferences, though generalizability beyond teleworkers and specific family configurations should be made with caution. Practically, interventions should prioritize sustaining resource-building and demand-reducing forms of job crafting, particularly for women, to mitigate FWC, while addressing the direct spillover from burnout to WFC in men through boundary norms, workload calibration, and recovery-oriented policies. Overall, the findings nuance expectations of a uniform loss cycle by demonstrating that its operation depends on conflict direction and gender.

## Data Availability

The raw data supporting the conclusions of this article will be made available by the authors, without undue reservation.
